# Effect of low dose aspirin application during pregnancy on fetal congenital anomalies

**DOI:** 10.1186/s12884-022-05142-8

**Published:** 2022-11-01

**Authors:** Si Sun, HongYang Qian, Congcong Li, Qiaohong Wang, Aimin Zhao

**Affiliations:** 1 Department of Obstetrics and Gynecology, School of Medicine, Ren Ji Hospital, Shanghai Jiao Tong University School of Medicine, Shanghai, 200127 China; 2 Department of Urology, Ren Ji Hospital, Shanghai Jiao Tong University School of Medicine, Shanghai, 200127 China

**Keywords:** Low dose aspirin, Congenital anomalies, Pregnancy, Teratogenicity

## Abstract

**Background:**

With the extensive use of aspirin in obstetrics and reproductive medicine, concerns of potentially related congenital anomalies have been raised in previous studies. However, there is a lack of evidence concerning the safety of application of aspirin during pregnancy in Chinese population, especially during the first trimester.

**Patients and methods:**

We retrospectively included a total of 2,763 patients with 2,856 fetuses (2670 singleton births and 93 pairs of twins), among whom 1,684 took low dose aspirin (LDA) during pregnancy (the LDA group) and the other 1,079 were not exposed to LDA (the control group). The primary outcome was the rate of fetal congenital anomalies, and was compared between the LDA group and the control group. We also conducted logistic regression to examine the potential risk factors of congenital abnormalities.

**Results:**

The average daily dose of LDA taken was 67.6 mg. The rate of congenital anomalies was comparable between the two groups, suggesting low teratogenicity of LDA application during pregnancy (3.3% vs. 2.8%; *P* = 0.421). The duration of LDA exposure and the time of LDA exposure showed no association with congenital anomalies. A previous history of fetal congenital anomalies was associated with an increased risk of the recurrence of congenital anomalies in the siblings (adjusted OR = 3.00, 95% CI: 1.00–8.60; *P* = 0.041).

**Conclusion:**

Exposure to LDA during pregnancy did not increase the risk of congenital anomalies in the fetus, suggesting that it was safe to apply LDA during pregnancy. A history of previous fetal abnormalities was found to be an independent risk factor of congenital anomalies. Our study suggests that LDA can be safely applied during pregnancy without increasing risks of congenital anomalies.

**Supplementary Information:**

The online version contains supplementary material available at 10.1186/s12884-022-05142-8.

## Introduction

Aspirin has been extensively used in obstetrics and reproductive medicine in recent years. In pregnancy-associated diseases, low dose aspirin (LDA) has been employed as an effective prophylaxis and treatment medication. Patients with a high risk of preeclampsia are recommended to receive LDA between 12 and 28 weeks of gestation [1]. Also, antiplatelet therapy with LDA can improve pregnancy outcomes in patients undergoing recurrent pregnancy loss with thrombophilia [2].

With extensive use of aspirin during pregnancy, it is of paramount importance to explore whether aspirin, especially LDA as a commonly used therapy in clinical practice, could cause congenital abnormalities. The question has been explored by many previous studies, with contradictory findings. In animal experiments, researchers found that high-dose aspirin administration in the first and second trimester was associated with various congenital anomalies, including cranial spina bifida, diaphragmatic hernia, gastroschisis, skeletal deformity, heart malformation, cleft lip and palate, and decreased virilization [3, 4]. In human studies, a possible association between aspirin use during the first trimester and fetal gastroschisis was reported [5–7], as well as the association of application in early and mid-pregnancy with cryptorchidism [8]. In addition, concerns were also raised about the effect of cyclooxygenase inhibitor on premature closure of ductus arteriosus [9], leading to worries about the potential risk of aspirin use in the last trimester. By contrast, recent systematic reviews found that LDA was not associated with an increased risk of congenital abnormalities [10, 11]. One RCT study enrolling 1,288 women, among whom 615 started using LDA before pregnancy and kept taking the medication throughout the whole pregnancy, suggested that exposure to LDA during pregnancy did not increase the risk of congenital malformations [12]. A recent meta-analysis included 8 RCTs (7564 participants assigned to LDA less than 14 weeks of gestation and 7670 serving as controls) and the results suggested no evidence of safety concerns regarding LDA teratogenicity [13]. However, LDA exposure of less than 14 weeks was selected as the criterion to delineate the first trimester rather than the embryonic period which demonstrates the critical window of teratogenicity. As a result, the maternal and fetal safety of LDA use during pregnancy needs further exploration.

It is noteworthy that aspirin may have different effects in different ethnic groups due to the difference in genetic architecture of cyclooxygenase-1, platelet glycoprotein and purine receptor [14]. However, to the best of our knowledge, no study has ever been conducted to examine the effect of exposure to LDA during pregnancy, especially during early pregnancy, in the Chinese population.

In the present study, we compared the rate of fetal congenital anomalies between patients with and without LDA exposure, and examined the potential risk factors of congenital anomalies.

## Patients and methods

### Study subjects

In this study, we retrospectively included patients aged 20–49 years old who attended the reproductive immunology and obstetric clinics in Ren ji Hospital, Shanghai Jiao Tong University, School of Medicine between November 1, 2017 and October 31, 2019, and had confirmed pregnancy outcomes. Patients were excluded from the study if they satisfied any of the following conditions: 1) had a history of taking other category D or X drugs as classified by the Food and Drug Administration or unauthorized drugs without identifiable composition; 2) had a history of taking high dose aspirin during pregnancy (> 150 mg/d); 3) had a history of exposure to radiation, chemicals, alcohol, or maternal smoking during pregnancy; 4) had a history of vaccination during pregnancy; 5) had suffered from upper respiratory tract infection or fever during early pregnancy; 6) had teratogenic pathogen infection during pregnancy; 7) had severe medical diseases; 8) underwent an early pregnancy loss without any morphological abnormality of the fetus; or 9) did not have a regular prenatal check-up.

We collected the following information of the included patients by retrieving their medical data and telephone follow-up: basic demographics, all medications received during pregnancy (including type of medication, time and dosage), pregnancy outcomes and neonatal conditions. The primary outcome was the rate of fetal congenital malformations.

### Clinical tests

From the 12th week of gestation, screening for fetal morphology and organ development was conducted every four weeks using ultrasound. The diagnosis of fetal congenital abnormalities was based on ultrasound or MRI examinations as appropriate during the pregnancy, karyotype analysis from amniotic fluid, and postpartum examinations. Neonatal examinations were performed by two senior neonatal doctors. Fetal congenital anomalies were classified based on birth defects categories of the National Maternal and Child Health Surveillance of China [15], coded according to the International Classification of Diseases (ICD-10), with specific details described and recorded.

### Statistical analyses

Categorical variables were reported as numbers and percentage. For continuous data, we reported mean ± standard deviation if a variable followed a normal distribution, and otherwise as median (interquartile range, IQR). Normality test was done using Kolmogorov–Smirnov test. Baseline characteristics of the different groups were compared by using independent sample t-test, Mann–Whitney U test, Chi-square test and Fisher’s exact test, as appropriate. To assess the association between congenital abnormalities and the clinical variables, we also conducted multivariable logistic regression which included variables that were significant in univariable logistic regression. All statistical analyses were performed using SPSS version 22.0 software package (IBM Corp., Armonk, NY, USA). All hypothesis tests were two-sided, and *P* < 0.05 was considered statistically significant.

## Results

**Fig. 1:**
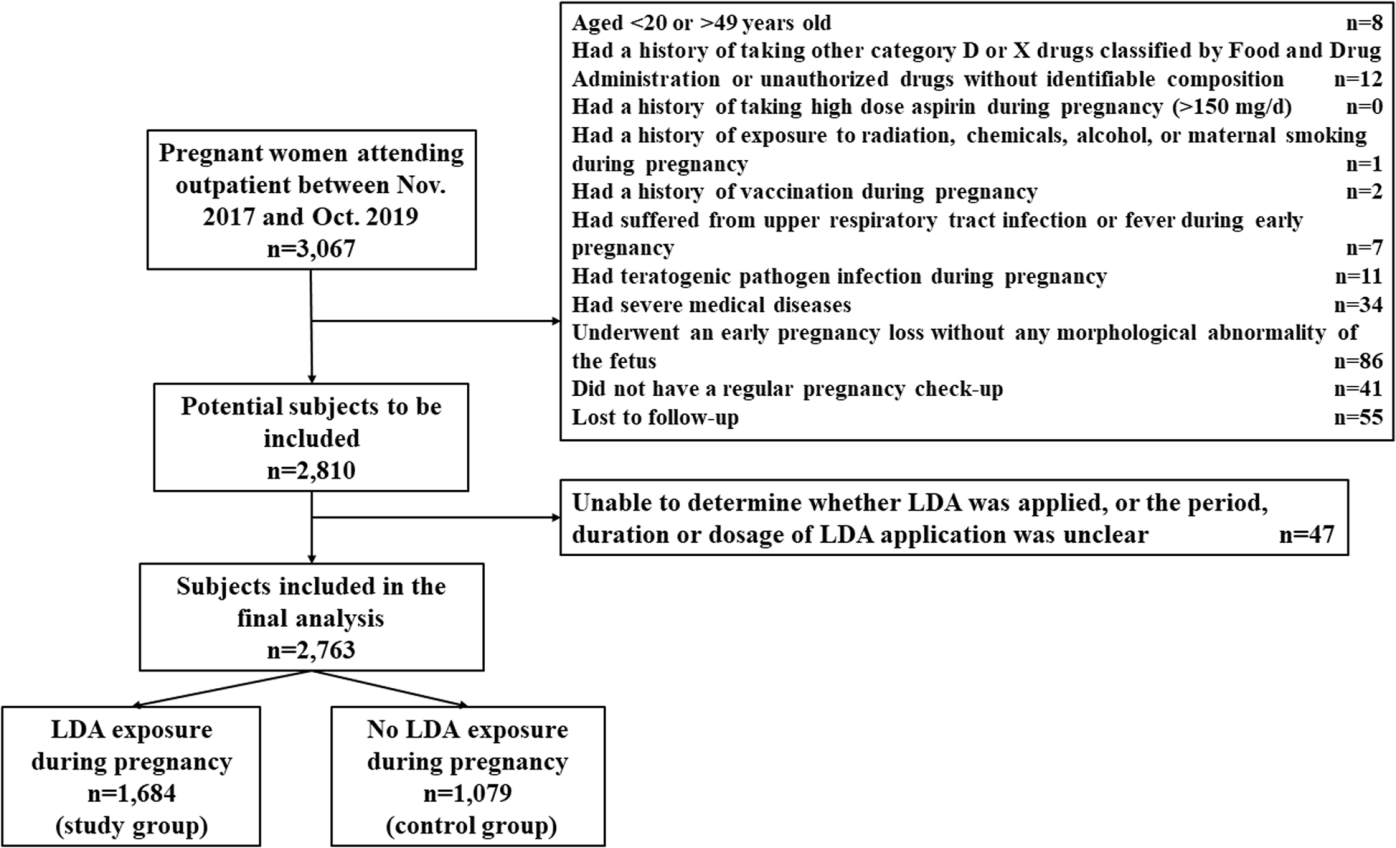
Selection process of subjects

A total of 2,763 patients with 2,856 fetuses (2670 singleton births and 93 pairs of twins) were included. Among them, 1,684 patients had taken LDA during pregnancy and the other 1,079 who were not exposed to LDA served as the controls. In the LDA group, average daily dose of LDA taken was 67.6 mg. And 1,543 (91.6%) patients were exposed to LDA in early pregnancy, 1,658 (98.5%) in the second trimester, 1,622 (96.3%) in the third trimester, and 1,494 (88.7%) throughout the pregnancy. The process of selecting eligible subjects is shown in Fig. 1.

Comparison of the general characteristics between the two groups was shown in Table 1. Patients in the LDA group were older (32 [29, 35] vs. 30 [28, 33]; *P* < 0.001), had higher primipara rate (87.8% vs. 72.0%; *P* < 0.001) and more previous spontaneous abortions (2 [0, 3] vs. 0 [0, 0]; *P* < 0.001), and were more likely to have chronic hypertension (1.7% vs. 0%; *P* < 0.001) and rheumatic diseases (10.2% vs. 3.4%; *P* < 0.001). We found no significant difference in the proportion of diabetes and thyroid diseases between the two groups.

**Table 1:** Comparison of the baseline characteristics between the two groups

	**LDA group (*n* = 1,684)**	**Control group (*n* = 1,079)**	***P* value**
Age, median (IQR) years	32 (29, 35)	30 (28, 33)	< 0.001
BMI, median (IQR) kg/m^2^	21.3 (19.7, 23.0)	21.3 (19.2, 23.2)	0.267
Nulliparas, n (%)	1478 (87.8)	755 (72.0)	< 0.001
Number of previous spontaneous abortion, median (IQR)	2 (0, 3)	0 (0, 0)	< 0.001
History of early miscarriage, n (%)	1198 (71.1)	103 (9.5)	< 0.001
History of late miscarriage, n (%)	183 (10.9)	13 (1.1)	< 0.001
History of still birth, n (%)	86 (5.1)	8 (0.7)	< 0.001
History of fetal congenital anomalies, n (%)	41 (2.4)	3 (0.3)	< 0.001
Complications			
Hypertension, n (%)	28 (1.7)	0	< 0.001
Diabetes, n (%)	3 (0.2)	0	0.427
Rheumatic disease, n (%)	172 (10.2)	37 (3.4)	< 0.001
Thyroid disease, n (%)	31 (1.8)	23 (2.1)	0.59

*Abbreviations:* *BMI* body mass index, *LDA* low dose aspirin, *IQR* inter-quartile range

The rate of fetal abnormalities in the LDA group and the control group was 3.3% and 2.8%, respectively, with no significant difference (*P* = 0.421, Table 2). Additionally, no significant differences were observed in the rate of each specific anomality between the two groups. When specifically comparing patients who had taken LDA during the first trimester with the controls, we found no significant difference in the rate of congenital anomalies (2.9% [45/1,543] vs. 2.8% [30/1,079]; *P* = 0.837).

**Table 2:** Comparison of fetal congenital abnormalities between the two groups

	**LDA group (*n* = 1,684)**	**Control group (*n* = 1,079)**	***P* value**
Fetal congenital abnormalities, n (%)	56 (3.33)	30 (2.78)	0.421
Anencephaly Q00, n (%)	0	0	NA
Spina bifida Q05, n (%)	0	0	NA
Encephalocele Q01, n (%)	2 (0.12)	0	0.524
Congenital hydrocephalus Q03, n (%)	0	0	NA
Cleft palate Q35, n (%)	2 (0.12)	0	0.524
Cleft lip Q36, n (%)	1 (0.06)	1 (0.09)	1
Cleft lip with cleft palate Q37, n (%)	2 (0.12)	0	0.524
Small ears (including earless) Q17.2, Q16.0, n (%)	1 (0.06)	0	1
Other abnormalities of the external ear Q17, n (%)	4 (0.24)	0	0.276
Esophagus atresia or stenosis Q39, n (%)	0	0	NA
Rectal and anal atresia or stenosis (including anus) Q42, n (%)	3 (0.18)	0	0.427
Hypospadias Q54, n (%)	1 (0.06)	1 (0.09)	1
Bladder exstrophy Q64.1, n (%)	0	0	NA
Clubfoot Q66.0, n (%)	1 (0.06)	2 (0.19)	0.697
Polydactyly Q69, n (%)	5 (0.30)	2 (0.19)	0.856
Syndactyly Q70, n (%)	3 (0.18)	1 (0.09)	0.949
Shortened limbs (including ectrodactyly and cleft hand/foot)			
Upper limb Q71, n (%)	0	1 (0.09)	0.391
Lower limb Q72, n (%)	0	0	NA
Congenital diaphragmatic hernia Q79.0, n (%)	1 (0.06)	0	1
Omphalocele Q79.2, n (%)	1 (0.06)	0	1
Gastroschisis Q79.3, n (%)	1 (0.06)	1 (0.09)	1
Conjoined twin Q89.4, n (%)	0	0	0
Down syndrome (trisomy 21) Q90, n (%)	0	0	0
Congenital heart diseases Q20-26, n (%)	19 (1.13)	13 (1.20)	0.854
Others, n (%)	12 (0.71)	8 (0.74)	0.930

*Abbreviations:* *LDA* low dose aspirin, *NA* not available

Note: If a fetus had multiple malformations, he/she was included into different categories separately

Using univariable logistic regression, we found that the number of previous spontaneous abortions (OR = 1.15, 95% CI: 1.01–1.31, *P* = 0.040) and a history of fetal malformations (OR = 3.22, 95% CI: 1.12–9.20, *P* = 0.029) were associated with the risk of fetal congenital abnormalities. We did not find a significant association of the other factors, such as duration of LDA exposure and time of LDA exposure, with the risk of fetal congenital abnormalities (Table 3). In the multivariable logistic regression, a history of fetal malformations remained statistically significant (OR = 3.00, 95% CI: 1.00–8.60; *P* = 0.041), but not the number of previous abortions (OR = 1.14, 95% CI: 1.00–1.31; *P* = 0.052; Table 4).

**Table 3:** Univariable logistic regression of fetal congenital abnormalities

Variables	OR	95% CI	*P* value
Age	1.01	0.96–1.07	0.640
BMI	0.94	0.86–1.02	0.124
Number of previous spontaneous abortions	1.15	1.00–1.31	0.040
History of previous preterm births	1.36	0.18–10.17	0.766
History of fetal congenital anomalies	3.22	1.12–9.20	0.029
Diabetes mellitus	1.09	0.64–1.88	0.745
Twin pregnancy	1.82	0.72–4.59	0.208
LDA exposure during pregnancy	1.20	0.77–1.89	0.421
Duration of LDA exposure	1.00	0.99–1.00	0.761
Time of exposure to LDA during pregnancy			
Exposure during the first trimester	0.86	0.56–1.33	0.505
Exposure during the second trimester	1.07	0.69–1.67	0.761
Exposure during the third trimester	0.80	0.52–1.23	0.316
Exposure to prednisone during pregnancy	0.76	0.49–1.19	0.233

*Abbreviations:* *OR* odds ratio, *CI* confidence interval, *BMI* body mass index, *LDA* low dose aspirin

**Table 4:** Multivariable logistic regression of fetal congenital abnormalities

Variables	OR	95% CI	*P* value
Number of previous spontaneous abortions	1.14	1.00–1.31	0.052
History of fetal congenital anomalies	3.00	1.04–8.60	0.041

*Abbreviations:* *OR* odds ratio, *CI* confidence interval

## Discussion

In this study, we explored the safety of application of LDA during pregnancy in Chinese population by retrospectively analyzing pregnancy outcomes of a cohort of pregnant patients with or without LDA exposure. We found that LDA exposure during pregnancy did not increase the risk of congenital anomalies, regardless of the duration of exposure and the time of exposure. To the best of our knowledge, this is the first study on the safety of LDA usage during pregnancy in the Chinese population.

While the pathogenesis of congenital anomalies is complex and remains largely unknown, it is hypothesized that congenital anomalies may be related with multiple factors, including chromosomal abnormalities, single-gene disorders, maternal inner environment, and external environmental factors such as chemicals and radiations. Previous research [16] suggested that 65% to 75% of birth defects were due to unknown causes with suspected polygenic and multifactorial etiologies. Single-gene disorders (15%–20%) and chromosomal abnormalities (5%) are the most common genetic etiologies. The remaining 10% of birth defects arose from environmental exposures, including medications, radiation, hyperthermia, chemical exposure, uterine abnormalities, maternal medical conditions, substance abuse and infection [16].

In early animal experiments, exposure to high dose aspirin during early and mid-pregnancy was found to be associated with a variety of congenital abnormalities, including craniorachischisis (which is extremely rare in humans but is related to anencephaly, exencephaly, and spinal bifida), facial clefts, eye defects, gastroschisis, skeletal deformity, cleft lip and palate, and decreased virilization [3, 4, 17, 18]. However, as the dosage used in the animal experiments to cause teratogenesis was much higher than what was commonly used in clinical practice, the results could not be extrapolated to humans. While in human studies, it remains controversial whether aspirin exposure during pregnancy could lead to congenital abnormalities in the fetus. Some studies found that exposure to aspirin in early pregnancy could increase the risk of gastroschisis [5, 6], consistent with findings in a meta-analysis of 5 studies (OR = 2.37; 95% CI: 1.44–3.88; *P* = 0.0006) [7]. Kristensen et al. suggested that aspirin exposure during early and mid-pregnancy might affect the development of the fetal reproductive system and increase the rate of cryptorchidism [8]. However, none of these studies specifically considered other potentially influential drugs taken during the pregnancy, and the dosage of aspirin taken was not indicated either. By contrast, other studies reported opposite results. In a large prospective cohort study, researchers compared the rate of fetal congenital anomalies between patients exposed to aspirin during early pregnancy with different durations of exposure (duration of exposure ≥ 8 days was regarded as severe exposure) with the unexposed women, and found that aspirin exposure during early pregnancy was not associated with fetal congenital malformations, regardless of the duration [19]. Another case–control study based on the data of birth-defect registry suggested that maternal use of aspirin during the first trimester was not associated with increased risk of four specific congenital anomalies (neural-tube defects, exomphalos/gastroschisis, cleft lip and posterior cleft palate) in the offspring compared with nonusers of aspirin [20].

It should be noted that the dosage of aspirin was not indicated in these studies. Because nonsteroidal anti-inflammatory drugs are over-the-counter drugs in many countries, subjects included in these studies may contain a large number of women who have consumed moderate to high doses of aspirin. The different findings may be due to differences in study subjects, dosages of aspirin, periods of exposure and use of other influential drugs during the pregnancy.

In recent years, LDA has been found to be able to effectively prevent the onset of preeclampsia, and has gained increasing application in obstetrics. In the 2020 guideline for the Management of Gestational Hypertension and Preeclampsia issued by the American College of Obstetrics and Gynecology [1], it was recommended that patients at risk of preeclampsia should take LDA from 12 to 28 weeks of gestation (preferably before 16 weeks of gestation) until childbirth. Recent meta-analyses and systematic reviews enrolling trials of using LDA to prevent the onset of preeclampsia suggested no association of LDA application with an increased risk of congenital anomalies [10, 11]. However, it should be noted that most of the included studies started LDA treatment after the early pregnancy, with the initial time of LDA application ranging from 12 to 36 weeks of pregnancy, and few studies started LDA in the first trimester which is a critical period for most congenital anomalies. In this study, the majority of patients in LDA group underwent an exposure during the first trimester, which might further provide evidence on the safety of LDA in terms of teratogenicity.

Although our data revealed no significant effect of LDA on the risk of congenital anomalies, we found some other factors that may contribute to risk of congenital anomalies. For example, we found that a history of fetal abnormalities was significantly associated with a higher risk of congenital anomalies: patients who had a history of fetal abnormalities had a two-folds higher risk, compared with those without a history (OR = 3.22, 95% CI: 1.12–9.20, *P* < 0.05). Our findings were consistent with results obtained in previous studies [21, 22], which used population-based registry data on 872,493 singleton stillbirths, live births and terminations of pregnancy for fetal anomaly from 1985 to 2010 in UK and found that patients whose first pregnancy was affected by congenital anomaly had 2.5 times higher risk of fetal anomaly than those with a normal first pregnancies [22]. The recurrence risk was considerably elevated, especially for similar anomalies (RR = 23.80, 95% CI: 19.60–27.90; *P* < 0.0001), while for dissimilar anomalies, the increase in risk was more modest (RR = 1.40, 95% CI: 1.20–1.60; *P* = 0.001). Our results suggested a trend of higher risk of congenital anomalies with an increased number of previous spontaneous abortions. Because women with recurrent pregnancy loss may share similar etiologies with patients affected by fetal anomalies, it is possible that a history of recurrent pregnancy loss could pose potential risk to congenital anomalies, as demonstrated in our study. Therefore, women who have experienced adverse pregnancy outcomes should receive enhanced monitoring when they get pregnant again. Careful attention should be paid in obstetric examination of possible fetal congenital abnormalities during pregnancy, especially for those who have a history of fetal abnormalities or spontaneous abortions.

To the best of our knowledge, the present study represents the first to explore the safety of LDA application during first trimester in Chinese population. Limitations of the present study include its retrospective nature and the relatively small size combined with the low rate of congenital anomalies. Future studies with prospective designs and larger sample sizes are needed to validate our findings.

## Conclusion

Extensive use of aspirin in obstetrics and reproductive medicine has raised concerns over its safety profile. Our study shows that exposure to LDA during pregnancy did not increase the risk of congenital anomalies in the fetus, regardless of the duration of exposure and time of exposure. Also, we have found that a history of fetal abnormalities was an independent risk factor of fetal congenital anomalies. Our study suggests that LDA can be safely applied during pregnancy for prophylaxis and treatment without increasing risks of congenital anomalies. Further research with larger cohorts and prospective designs is needed.

## Supplementary Information

**Additional file 1.** The “raw datasets” file includes raw clinical data of all patients included in this study. As shown in the raw datasets, each patient has the detailed clinical data including age, body mass index (BMI), number of previous early miscarriage, number of previous late miscarriage, number of previous still birth, number of previous spontaneous abortions, history of preterm birth, history of fetal congenital anomalies, nulliparas, twin birth and etc. Also, the low dose aspirin (LDA) exposure during pregnancy including time and duration for each patient are well recorded in the spreadsheet. The complications before pregnancy are also recorded in the spreadsheet.

## Data Availability

The data generated or analyzed during this study are included in this published article and its supplementary information files.
